# Draft Genome Sequence of a Red-Pigmented *Janthinobacterium* sp. Native to the Hudson Valley Watershed

**DOI:** 10.1128/genomeA.01429-17

**Published:** 2018-01-04

**Authors:** Kelsey O’Brien, Gabriel G. Perron, Brooke A. Jude

**Affiliations:** a Pediatric Infectious Disease Division, Children’s Hospital of Philadelphia, Philadelphia, Pennsylvania, USA; b Reem-Kayden Center for Science and Computation, Program of Biology, Bard College, Annandale-on-Hudson, New York, USA

## Abstract

Water samples from the Hudson Valley watershed indicate that the area is host to many violacein-producing bacterial isolates. Here, we report the draft whole-genome sequence of *Janthinobacterium* sp. strain BJB412, an isolate lacking violacein production yet containing genes responsible for prodigiosin, biofilm production, and quorum sensing, like its purple-pigmented counterparts.

## GENOME ANNOUNCEMENT

*Janthinobacterium* spp. are aerobic motile Gram-negative bacteria that are commonly characterized by their production of a purple metabolite, violacein (1). This product of a five-gene *vio* operon (2) was recently linked to killing effects on an amphibian-specific fungus, *Batrachochytrium dendrobatidis* (3–6). Many *Janthinobacterium* strains possess quorum-sensing capabilities (7–9) to regulate phenotypes, such as violacein and biofilm production (9–12). The bacterial isolate in this study, BJB412, was cultured from the Hudson River watershed in New York and does not produce the purple violacein pigment characteristic of the genus. Instead, BJB412 is characterized by a vibrant red color, predicted to result from the production of prodigiosin, a pigment with antimicrobial properties (13). Interestingly, BJB412 was isolated from a water sample alongside violacein-pigmented colonies.

Genomic DNA extraction was completed with the Qiagen Gentra Puregene Yeast/Bact. kit using vendor-provided protocols. Paired-end Illumina libraries (150 bp) were prepared, and HiSeq sequencing using Illumina HiSeq 4000 was completed offsite (Wright Labs, Huntington, PA). Read assemblies were built using a modified version of a local pipeline described elsewhere (14). To this protocol, adapters and contaminants were removed, and reads were quality filtered with a *Q* score cutoff of 10 using BBDuk from the BBMap package version 37.50 (https://sourceforge.net/projects/bbmap). A draft assembly was built using SPAdes version 3.11.0 (15) (k-mers selected, 21, 33, 55, 77, 99, and 127). Contigs shorter than 500 bp or that comprised fewer than four reads were subsequently filtered out of the assembly. Assembly improvement was attempted using a combination of SSPACE and GapFiller (16–18).

Draft assembly of the whole genome yielded 78 contigs, with an *N*_50_ value of 333,942 bp. The genome of BJB412 is predicted to be 6,786,668 bp in length, which is comparable to that of other analyzed *Janthinobacterium* species. Interestingly, analysis revealed a G+C content of 67.16%, while most other published *Janthinobacterium* genomes have a G+C content ranging from 62 to 63% (12, 19, 20). The assembled contigs were annotated using a local pipeline running the Prokka genome annotation software (21), the RASTtk annotation software, via the PATRIC pipeline (22, 23), and the NCBI Prokaryotic Genome Annotation Pipeline (PGAP) (24). Annotations for BJB412 yielded an average of 5,932 coding sequences (CDSs). As expected, a violacein biosynthesis operon was not present in any annotation, while the sequences for the *pig* genes, which are responsible for prodigiosin production, were observed. Additionally, annotation involved genes that participate in the bacterial quorum-sensing cascade (*jqsA* and *qseC*) and genes related to cyclic-di-GMP (c-di-GMP) levels, biofilm production (*wspC*), chemotaxis-mediated biofilm dispersion (*bdlA*), and twitching motility (*pilT, pilJ, pilH,* and* pilG*) (25, 26). Related to the biofilm genotype, BJB412 displays an interesting colonial morphology distinct from all other *Janthinobacterium* isolates observed: the bacterial colonies are firmly embedded in the medium when cultured on 1.5% R2A agar.

BJB412 was found in the same aquatic community as violacein-producing bacterial strains. It is possible that the pigments produced by BJB412 work in association with violacein, potentially having additive killing effects on local pathogens. Future work aims to better understand how these bacterial genomes contribute to fungal remediation and their eventual therapeutic implementations.

### Accession number(s).

The whole-genome shotgun projects have been deposited at DDBJ/ENA/GenBank under accession number PDZP00000000. The version described in this paper is version PDZP01000000.
